# Diaphragm Muscle Weakness Following Acute Sustained Hypoxic Stress in the Mouse Is Prevented by Pretreatment with N-Acetyl Cysteine

**DOI:** 10.1155/2018/4805493

**Published:** 2018-02-19

**Authors:** Andrew J. O'Leary, Sarah E. Drummond, Deirdre Edge, Ken D. O'Halloran

**Affiliations:** ^1^Department of Physiology, School of Medicine, University College Cork, Cork, Ireland; ^2^Department of Physiology, Trinity Biomedical Sciences Institute, Trinity College Dublin, The University of Dublin, Dublin, Ireland

## Abstract

Oxygen deficit (hypoxia) is a major feature of cardiorespiratory diseases characterized by diaphragm dysfunction, yet the putative role of hypoxic stress as a driver of diaphragm dysfunction is understudied. We explored the cellular and functional consequences of sustained hypoxic stress in a mouse model. Adult male mice were exposed to 8 hours of normoxia, or hypoxia (FiO_2_ = 0.10) with or without antioxidant pretreatment (N-acetyl cysteine, 200 mg/kg i.p.). Ventilation and metabolism were measured. Diaphragm muscle contractile function, myofibre size and distribution, gene expression, protein signalling cascades, and oxidative stress (TBARS) were determined. Hypoxia caused pronounced diaphragm muscle weakness, unrelated to increased respiratory muscle work. Hypoxia increased diaphragm HIF-1*α* protein content and activated MAPK, mTOR, Akt, and FoxO3a signalling pathways, largely favouring protein synthesis. Hypoxia increased diaphragm lipid peroxidation, indicative of oxidative stress. FoxO3 and MuRF-1 gene expression were increased. Diaphragm 20S proteasome activity and muscle fibre size and distribution were unaffected by acute hypoxia. Pretreatment with N-acetyl cysteine substantially enhanced cell survival signalling, prevented hypoxia-induced diaphragm oxidative stress, and prevented hypoxia-induced diaphragm dysfunction. Hypoxia is a potent driver of diaphragm weakness, causing myofibre dysfunction without attendant atrophy. N-acetyl cysteine protects the hypoxic diaphragm and may have application as a potential adjunctive therapy.

## 1. Introduction

The diaphragm is the principal muscle of inspiration, an integral part of the thoracic “pump” musculature, responsible for effective lung ventilation. Diaphragm muscle weakness can present in respiratory disease, and it is a strong predictor of poor outcome in clinical patients, particularly those requiring mechanical ventilation [1]. Hypoxia (oxygen deficit) commonly presents as a feature in many acute respiratory conditions including acute respiratory distress syndrome, ventilator-induced lung injury, ventilator-associated lung injury, and acute lung injury, conditions where diaphragm muscle weakness is typically observed [2–6]. Interventions serving to prevent or suppress diaphragm muscle weakness in hypoxaemic respiratory patients could have substantially beneficial effects for patient outcome. Whereas this is an area of intense research, it appears that the potentially deleterious role of hypoxic stress in the development and manifestation of diaphragm muscle weakness has been overshadowed by other factors such as mechanical unloading (inactivity), injury, infection, and sepsis, despite the potential for significant cellular redox modulation during oxygen deficit, and the well-recognized dominant role of oxidative stress as a contributory factor in respiratory and skeletal muscle wasting in critically ill patients [7–12].

It is established that exposure to chronic sustained hypoxia results in respiratory myofibre atrophy and muscle weakness associated with a time-dependent progressive oxidative stress [13–16]. We recently demonstrated that acute sustained hypoxia (8 hours of exposure) is sufficient to weaken mouse diaphragm peak force-generating capacity by ~30% [17, 18]. Our finding provides a basis for the hypothesis that acute hypoxia-induced diaphragm dysfunction could be an underrecognized and yet major factor contributing to diaphragm weakness in the critical care setting [18], and perhaps during exposure to environmental hypoxia at high altitude [19–21].

It is recognized that antioxidants have considerable therapeutic efficacy in the treatment of respiratory-related disorders featuring redox imbalance and overt oxidative stress, such as acute lung injury, sepsis, chronic obstructive pulmonary disease, chronic bronchitis, lung fibrosis, and cystic fibrosis [22–30]. N-acetyl cysteine (NAC) is a free radical scavenger, which boosts the synthesis of the endogenous antioxidant glutathione as well as other antioxidant systems. Chronic NAC supplementation has been shown to prevent diaphragm muscle weakness in animal models of chronic sustained hypoxia [16] and chronic intermittent hypoxia [31, 32]. Of interest, acute respiratory distress syndrome patients are in a prooxidant state, which can be improved by NAC supplementation, by increasing glutathione, thiol molecules, and antioxidant defences [33]. The muscles of breathing, like other striated muscles, are highly dependent on redox balance for optimal function [15, 16, 34, 35]. We reasoned that acute hypoxic stress is detrimental to muscle function. If so, antioxidant intervention may alleviate acute hypoxia-induced diaphragm dysfunction, a finding which if it translates to humans could prove useful in the treatment of hypoxaemic respiratory patients in the clinic.

Therefore, we sought to further explore the cellular mechanisms underpinning acute hypoxia-induced diaphragm weakness and establish if pretreatment with NAC at the outset of acute hypoxic exposure prevents or ameliorates diaphragm muscle weakness. The aims of our study were to (1) confirm the deleterious effects of acute sustained hypoxia on mouse diaphragm force-frequency relationship; (2) determine the whole body integrative ventilatory and metabolic strategies adopted by mice during acute hypoxic exposure; (3) examine protein signalling pathways in diaphragm muscle, putatively affected by acute hypoxic stress; (4) examine gene expression changes in the diaphragm associated with atrophy and autophagy processes; (5) determine if 20S proteasome activity is increased by acute hypoxic stress in the diaphragm; (6) determine if diaphragm myofibre size and distribution are affected by acute hypoxia; (7) determine if acute hypoxia causes diaphragm oxidative stress; and (8) explore the efficacy of NAC pretreatment in ameliorating diaphragm functional responses to acute sustained hypoxia.

## 2. Methods

### 2.1. Ethical Approval

All protocols involving animals described in this study were performed under licence from the Irish Government in accordance with the National and EU legislation following institutional animal research ethics committee approval.

### 2.2. Animal Model

Models of acute hypoxia were generated using adult male C57BL6/J mice (Envigo, UK). Mice (~14 weeks of age) were placed in environmental plethysmography chambers (Buxco Ltd., USA), at room temperature, in which ambient oxygen levels were measured and adjusted to desired levels (gas analyzer, ML206, AD Instruments, UK). Mice were exposed to one, four, or eight hours of hypoxia (fraction of inspired oxygen, FiO_2_ = 0.10) or normoxia (FiO_2_ = 0.21) (*n* = 8 per group). An additional group of mice (*n* = 8) were each given a single injection of N-acetyl cysteine (NAC; 200 mg/kg; i.p.) immediately prior to 8 hours of hypoxia exposure.

### 2.3. Whole Body Plethysmography

Breathing parameters, including respiratory frequency (f_R_), tidal volume (V_T_), and minute ventilation (${\dot{V}}_{E}$) were measured on a breath-by-breath basis. O_2_ consumption ($\dot{V}O_{2}$) and CO_2_ production ($\dot{V}{CO}_{2}$) were monitored over the course of the gas exposure, serving as an index of whole body metabolism. Gas concentrations entering and leaving the plethysmography chambers were sampled using a gas analyzer (ML206, AD Instruments). The respiratory exchange ratio ($\dot{V}{CO}_{2}/\dot{V}O_{2}$) and the ventilatory equivalent for carbon dioxide (${\dot{V}}_{E}/\dot{V}{CO}_{2}$) were determined offline. Following 8 hours of gas exposure, animals were euthanized using a rising concentration of CO_2_ until narcosis, followed immediately by cervical dislocation to confirm euthanasia. Core body temperature was determined immediately postmortem, via insertion of a probe (TH-8 Thermalert, Physitemp Instruments, Inc., USA); body mass was recorded. Diaphragm muscles were quickly excised and either immediately used for functional analysis or immediately snap frozen in liquid nitrogen and stored at −80°C until further processing for molecular analysis.

### 2.4. Muscle Physiology: Experimental Setup

Experiments were performed on diaphragm muscle preparations derived from the 3 groups of mice: normoxia, hypoxia, and hypoxia + NAC. Once excised, diaphragm muscles were placed in a holding bath containing continuously gassed (95% O_2_/5% CO_2_) Krebs solution at room temperature. A longitudinal strip of diaphragm muscle with rib and central tendon intact was prepared and mounted vertically in a tissue holder by attaching the rib to a fixed hook and connecting the tendon to a force transducer (Aurora Scientific, USA) for the assessment of isometric contractile parameters. The preparation was housed in a tissue bath of Krebs solution at 35°C, gassed with 95% O_2_/5% CO_2_.

### 2.5. Muscle Physiology: Experimental Protocol

Muscle preparations were set to optimal length by adjusting the length of the tissue bundle between repeated twitch contractions until peak twitch force was determined. The relationship between stimulation frequency and tetanic force generation (force-frequency relationship) was assessed by stimulating the muscle strip at increasing stimulation frequencies from 10 Hz to 160 Hz and recording the force generated at each frequency. Forces recorded at each frequency were then normalized to the estimated cross-sectional area of the muscle tissue bundle, and data were expressed as specific force in N/cm^2^.

### 2.6. Gene Expression: RNA Extraction and Preparation

Total RNA was extracted from 20–70 mg of frozen diaphragm tissue homogenized using a general laboratory homogenizer (Omni-Inc., USA) in the presence of Tripure Isolation Reagent (Roche Diagnostics Ltd., UK). The manufacturer's instructions were followed with the addition of a chloroform wash step during phase separation. Next, RNA was treated with TURBO DNA-free Kit (Life Technologies, Bio-Sciences, Ireland) in accordance with the manufacturer's instructions. The quantity and purity of RNA were assessed using a Nanodrop 1000 (Thermo Scientific, USA) and spectrophotometry. The integrity of RNA was assessed by visualization of clear 18S and 28S ribosomal RNA bands using an agarose gel electrophoresis system (E-gel, Life Technologies).

### 2.7. Gene Expression: Reverse Transcription

Diaphragm muscle RNA was reverse transcribed using Transcriptor First Strand cDNA Synthesis Kit (Roche Diagnostics Ltd.) in accordance with the manufacturer's instructions.

### 2.8. Gene Expression: qRT-PCR

cDNA was amplified using Realtime ready Catalog or Custom Assays (Roche Diagnostics Ltd.) and Fast Start Essential DNA Probe Master (Roche Diagnostics Ltd.) in 20 *μ*l reactions (5 *μ*l cDNA and 15 *μ*l master mix) using the LightCycler 96 (Roche Diagnostics Ltd.) on 96-well plates, in accordance with the manufacturer's instructions. All reactions were performed in duplicate and reverse transcriptase negatives, RNA negatives, and cDNA negatives (no template), and plate calibrator controls were used on every plate. Diaphragm gene expression was normalized to a reference gene, *hprt1*, to compensate for variations in input amounts of RNA/cDNA and the efficiency of reverse transcription. In preliminary studies, several candidate reference genes were screened, and in consideration of temporal and gas (hypoxia) exposures, *hprt1* was found to be most stable. The relative expression of genes was calculated using the ΔΔCT method, that is, normalized expression of the gene of interest to that of the reference gene, with changes in expression displayed as a fold change relative to the control group.

### 2.9. Cell Signalling: Protein Extraction and Quantification

Frozen muscle samples were removed from storage at −80°C, weighed and homogenized on ice in ice-cold 2.5% *w*/*v* modified radioimmunoprecipitation assay (RIPA) buffer (1X RIPA, deionized H_2_O, 200 mM sodium fluoride, 100 mM phenylmethylsulfonylfluoride (PMSF), 1X protease inhibitor cocktail, and 2X phosphatase inhibitor cocktail) using a general laboratory homogenizer (Omni-Inc., USA). Following 20-minute lyse time on ice, with vortexing every 4 minutes, samples were centrifuged in a U-320R centrifuge (Boeckel & Co, Germany) at 14,000 RPM at 4°C for 20 minutes to separate insoluble cellular fractions from the protein homogenates. The protein containing supernatant was separated from the insoluble pellet in each sample, and these were stored at −80°C. The pellets were discarded.

The protein concentration of each sample was determined using a bicinchoninic acid (BCA) protein quantification assay (Pierce Biotechnology, (Fisher Scientific), Ireland) as per the manufacturer's instructions, at a dilution of 1 : 3.

### 2.10. Cell Signalling: Hypertrophy, Atrophy and HIF Signalling, and Protein/Phosphoprotein Content Assays

Cell signalling assays were performed using an Akt signalling panel–phospho-Akt, phospho-p70S6K, phospho-S6RP, and phospho-GSK-3*β*; Phospho-/Total mTOR; Phospho-FOXO3a; a MAP Kinase phosphoprotein panel–phospho-p-38, phospho-ERK1/2, and phospho-JNK; and total HIF-1*α* (MesoDiscovery, USA).

The assays measure the protein or phosphorylated protein content of signalling proteins listed above. The sandwich immunoassays were either in multiplex format (Mesoscale Discovery, USA) allowing the measurement of up to 4 proteins/phosphoproteins in one well, or singleplex assay which allowed measurement of the content of one protein/phosphoprotein per well. The assay was carried out in accordance with the manufacturer's instructions. Briefly, samples were loaded onto the 96-well plate and incubated to allow the proteins of interest to be captured by their specific capture antibodies. Each well was then washed and incubated in a detection antibody solution. The detection antibodies, specific for each protein of interest, are conjugated with an electrochemiluminescent compound, or tag. The detection antibodies bind to the captured proteins of interest, which are bound to their specific capture antibodies, on the distinct spots on the bottom surface of the well. This completes the antibody-protein-antibody sandwich. The well was then washed again, and read buffer was added, creating the correct chemical environment for electrochemiluminescence. The plate was loaded into the MSD SECTOR Imager (a specialized spectrophotometer from Mesoscale) where a voltage is rapidly applied to the distinct working electrodes on the plate spots resulting in the tags conjugated to the antibodies attached to those electrodes emitting light, which is read by the imager at 620 nm. The intensity of this emitted light from each spot, separated both temporally and spatially, provides a quantitative measurement for each protein of interest in the sample. This assay was performed on diaphragm samples from control, hypoxia, and hypoxia + NAC groups. A series of dilutions with muscle sample was also assayed as a control to demonstrate increased luminescence with increased protein content added to the well for each assay.

### 2.11. Thiobarbituric Acid Reactive Substances (TBARS) Assay

TBARS are degradation products of fats which are routinely used as a marker of lipid peroxidation, an indirect measurement of oxidative stress. Malondialdehyde (MDA) of a known concentration was used to generate a standard curve. Next, 50 *μ*l of thiobarbituric acid (TBA, 50 mM) was added to 50 *μ*l of diaphragm muscle homogenate (as described above in section 2.9), and the solution was incubated for 60 mins at 97°C. Samples were subsequently cooled on ice, and 75 *μ*l of methanol : 1 mM NaOH (91 : 9) was added to the solution. Samples were then centrifuged at 704*g*, and 70 *μ*l of the resultant supernatant was added per well in a black 96-well plate. The plate was read in a SpectraMax-M3 spectrophotometer (Molecular Devices, USA) using 523/553 excitation/emission settings, and data were compared with standards. Data are expressed as nM TBARS per mg of protein in the sample, determined by BCA assay.

### 2.12. Proteasome Activity Assay

Chymotrypsin-like 20S proteasome activity was measured via fluorescence in a spectrophotometric assay as per the manufacturer's instructions (Abcam, UK) using a Spectramax M3 spectrophotometer (Molecular Devices, USA). The assay employs a peptide substrate tagged to AMC. In the presence of proteasome activity, the AMC tag is released and fluoresced. The kit includes a positive control in the form of Jurkat cell lysate with high proteasome activity and a specific proteasome inhibitor MG-132, which suppresses all proteolytic activity attributed to proteasomes, thus allowing the differentiation of proteasome activity from other protease activity in the sample. This assay was performed on diaphragm samples from normoxia, hypoxia, and hypoxia + NAC groups on a white 96-well plate; all samples and positive controls were assayed with and without the proteasome inhibitor. The samples, positive controls, and standards were added to the 96-well plate. Inhibitor was added to assigned wells (an equal volume of assay buffer was added to the uninhibited wells), and proteasome substrate was added to all wells except the standards. The plate was then incubated at 37°C in the spectrophotometer (protected from light) for one hour while fluorometric readings were made kinetically over that time at Ex/Em = 350/440 nm. There was a slight nonlinearity to the reaction kinetics at the beginning due to the lag time it takes for the reaction to mix and warm to 37°C. Readings were made from the linear range of the reaction. Nonproteasome activity was then subtracted from total activity to give proteasome activity. One unit of proteasome activity is defined as the amount of proteasome which generates 1 nmol of AMC per minute at 37°C.

### 2.13. Muscle Immunohistochemistry

Sections of hemidiaphragm from control (*n* = 6) and hypoxia (*n* = 6) mice were mounted on cubes of liver to facilitate subsequent tissue sectioning. Samples were coated in optimum cutting temperature (OCT; VWR International, Dublin, Ireland) embedding medium and then quickly frozen in isopentane (Sigma Aldrich, Wicklow, Ireland) cooled in liquid nitrogen. Tissue samples were subsequently stored at −80°C for structural analysis at a later time. Serial transverse muscle sections (10 *μ*m) were generated using a cryostat (Leica CM3050; Leica Microsystems, Nussloch, Germany) at −22°C and mounted on polylysine-coated glass slides (VWR International, Dublin, Ireland). Slides were immersed for 15 minutes in phosphate-buffered saline (PBS, 0.01 M) containing 1% bovine serum albumin (BSA). After PBS washes (3 × 5 minute), slides were next immersed for 30 minutes in PBS containing 5% normal goat serum (Sigma Aldrich, Wicklow, Ireland). Slides then underwent PBS washes (3 × 5 minute) prior to the application of the primary antibody (rabbit anti-laminin, 1 : 200; Sigma Aldrich, Wicklow, Ireland), diluted in PBS and 1% BSA. Slides were then incubated overnight at 4°C in a humidity chamber. The following day, slides were washed with PBS (3 × 5 minutes) before application of the secondary antibody (FITC-conjugated goat anti-rabbit; 1.250, Sigma Aldrich, Wicklow, Ireland), which was diluted in PBS and 1% BSA. Slides were incubated for 1 hour at room temperature in the dark.

Using an Olympus BX51 microscope and an Olympus DP71 camera, muscle sections were viewed at ×10 magnification and images were captured for analysis. For each animal, several images from separate muscle sections were captured for analysis. We employed a square test frame of 600 × 600 *μ*m, with inclusion and exclusion boundaries, which was placed randomly over each image [32]. To determine the size of diaphragm muscle fibres, the individual fibre perimeters were manually highlighted using Image J software allowing the determination of fibre cross-sectional area and minimal Feret's diameter. The coefficient of variation of these parameters was also computed. Data generated from multiple sections were first averaged for each animal before computing group means. Relative frequency histograms were constructed to illustrate the distribution of muscle fibre size in sections from control and hypoxia mice.

### 2.14. Data and Statistical Analysis

GraphPad Prism (GraphPad Software Inc., USA) was used to perform statistical analysis. Following tests for normality and equal variance in the data sets, the Student *t*-tests were performed for comparisons between two groups: normoxia and hypoxia. For multiple groups, one-way or two-way ANOVA (with Tukey's and Bonferroni post hoc tests, resp.) were used as appropriate. Statistical significance was taken at the level of *p* < 0.05.

## 3. Results

### 3.1. Diaphragm Force

**Figure 1:**
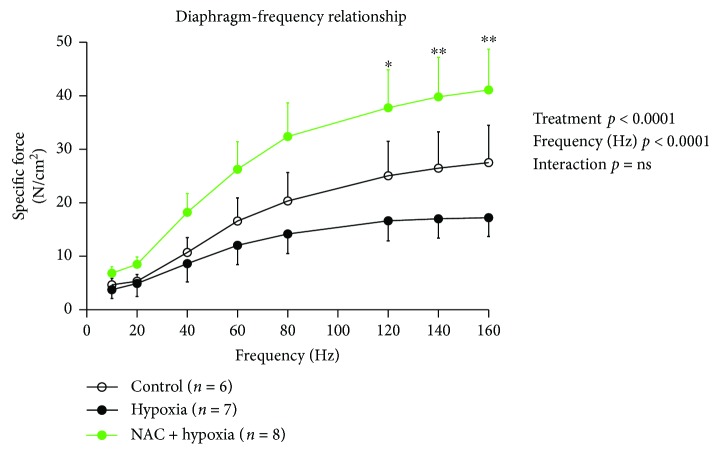
Mouse diaphragm muscle force-frequency relationship following 8 hours of exposure to normoxia (control), hypoxia, or hypoxia + NAC. Diaphragm-specific force (mean ± SEM) expressed as force per unit cross-sectional area of muscle (N/cm^2^) as a function of stimulation frequencies ranging between 10 and 160 Hz; *n* = 6–8 per group. Data were compared statistically by two-way (gas treatment × stimulation frequency) ANOVA; ^∗^*p* < 0.05 and ^∗∗^*p* < 0.01. Bonferroni's post hoc multiple comparisons test hypoxia + NAC versus hypoxia.

The mouse diaphragm force-frequency relationship following exposure to 8 hours of normoxia, hypoxia, and hypoxia + NAC is displayed in Figure 1. Specific force-generating capacity of the diaphragm muscle increased as a function of stimulation frequency (10–160 Hz; frequency: *p* < 0.0001, two-way ANOVA). Specific force generation over the range of stimulation frequencies tested was significantly lower in the hypoxia group compared with the normoxia group (gas: *p* = 0.0112); this weakness was most prominent at higher stimulation frequencies. A single i.p. injection of NAC prior to hypoxic exposure completely prevented hypoxia-induced diaphragm muscle weakness (*p* < 0.0001 versus hypoxia). Indeed, the hypoxia + NAC group developed the highest forces, exceeding control values over the range of stimulation frequencies (Figure 1).

### 3.2. Metabolism and Breathing

**Figure 2:**
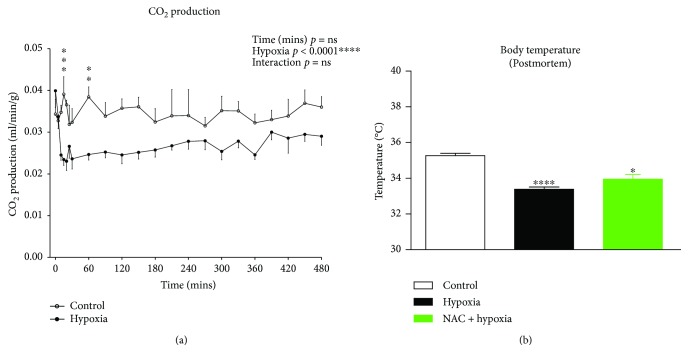
(a) Carbon dioxide production over 8 hours of breathing in mice exposed to normoxia (control) or hypoxia. Carbon dioxide production (mean ± SEM) in mice expressed as ml per minute per gram body mass (ml/min/g) over the 480-minute (8 hour) period of exposure to either normoxia (FiO_2_ = 0.21, *n* = 6) or hypoxia (FiO_2_ = 0.10, *n* = 7); two-way (time × gas treatment) ANOVA and Bonferroni's multiple comparisons test. ^∗∗∗^*p* < 0.001 and ^∗∗^*p* < 0.01 compared with corresponding normoxic values. (b) Postmortem body temperatures in mice following 8 hours of exposure to normoxia (control), hypoxia, or hypoxia + NAC. Postmortem body temperatures (mean ± SEM) of mice exposed to 8 hours of normoxia (FiO_2_ = 0.21, *n* = 6), hypoxia (FiO_2_ = 0.10, *n* = 7), or hypoxia + NAC (200 mg/kg i.p. at FiO_2_ = 0.10, *n* = 4). ^∗∗∗∗^*p* < 0.0001, unpaired *t* test compared with control; ^∗^*p* = 0.0105, unpaired *t* test with Welch's correction compared with control.

CO_2_ production over 8 hours of exposure to normoxia (control) and hypoxia is shown in Figure 2(a). CO_2_ production was significantly lower in the hypoxia group compared with the normoxia group (two-way ANOVA; gas: *p* < 0.0001) over the duration of the gas exposures, revealing a hypometabolic response to sustained hypoxia in mice.

Postmortem body temperature following gas treatments in all 3 groups is shown in Figure 2(b). Compared with mice in the normoxia group, body temperatures were lower in mice exposed to hypoxia (*p* < 0.0001, unpaired *t* test) and hypoxia + NAC (*p* = 0.0105, unpaired *t* test with Welch's correction). There was no difference in body temperature between hypoxia and hypoxia + NAC groups.

**Figure 3:**
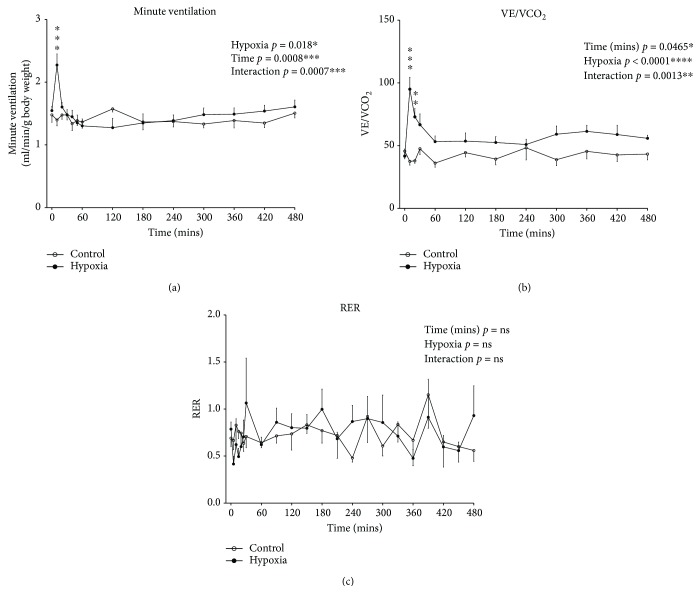
Minute ventilation (${\dot{V}}_{E}$), ventilatory equivalent for carbon dioxide (${\dot{V}}_{E}/\dot{V}{CO}_{2}$), and respiratory exchange ratio (RER; $\dot{V}{CO}_{2}/\dot{V}O_{2}$) during 8 hours of exposure to normoxia (control) or hypoxia. (a) Minute ventilation (mean ± SEM) expressed as ml per min per gram body mass (ml/min/g) over the 480-minute (8 hour) period of exposure to either normoxia (FiO_2_ = 0.21, *n* = 6) or hypoxia (FiO_2_ = 0.10, *n* = 7); two-way ANOVA (time × gas treatment) and Bonferroni's multiple comparisons test, ^∗∗∗^*p* < 0.001 compared with corresponding normoxic value. (b) Ventilatory equivalent ratio (mean ± SEM) in mice over the 480-minute (8 hour) period of exposure to either normoxia (FiO_2_ = 0.21, *n* = 6) or hypoxia (FiO_2_ = 0.10, *n* = 7); two-way ANOVA (time × gas treatment) and Bonferroni's multiple comparisons test, ^∗∗∗^*p* < 0.001 and ^∗∗^*p* < 0.01 compared with corresponding normoxic value. (c) Respiratory exchange ratio (mean ± SEM) in mice over the 480-minute (8 hour) period of exposure to either normoxia (FiO_2_ = 0.21, *n* = 6) or hypoxia (FiO_2_ = 0.10, *n* = 7); two-way ANOVA (time × gas treatment).

Figure 3(a) shows mouse respiratory ${\dot{V}}_{E}$, a product of f_R_ and V_T_, throughout the duration of normoxia (control) and hypoxia. ${\dot{V}}_{E}$ was increased (two-way ANOVA; gas: *p* < 0.001) after 10 mins of hypoxia compared with normoxia, but returned to levels equivalent to normoxia by 20 mins and remained at levels similar to normoxia for the remainder of the 8-hour exposure to hypoxia. Thus, beyond the initial transient hypoxic ventilatory response, ventilation was largely unchanged during hypoxic exposure in mice.

The ventilatory equivalent for carbon dioxide (${\dot{V}}_{E}/\dot{V}{CO}_{2}$) over 8 hours of exposure to normoxia (control) and hypoxia is shown in Figure 3(b). ${\dot{V}}_{E}/\dot{V}{CO}_{2}$ was significantly increased in the hypoxic group upon gas exposure compared with the control group, and it remained elevated over the course of the 8 hours of gas exposure (two-way ANOVA; gas: *p* < 0.0001), indicative of the development of a hypoxia-induced hyperventilation (arising due to hypometabolism with maintained ventilation).

Figure 3(c) shows the respiratory exchange ratio ($\dot{V}{CO}_{2}/\dot{V}O_{2}$) over 8 hours of exposure to normoxia (control) and hypoxia. There was no significant effect of exposure to hypoxia on $\dot{V}{CO}_{2}/\dot{V}O_{2}$ over the course of the 8 hours of gas exposure compared with control.

### 3.3. HIF-1*α*: Gene Expression and Protein Content

**Figure 4:**
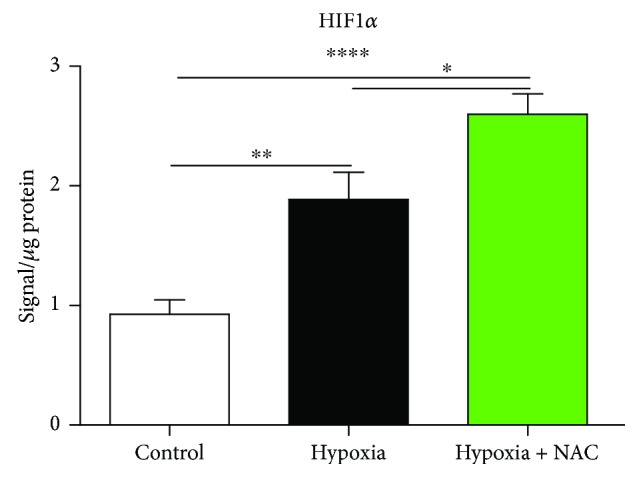
Mouse diaphragm HIF-1*α* protein content following 8 hours of exposure to normoxia (control), hypoxia, or hypoxia + NAC. Diaphragm HIF-1*α* protein content (mean ± SEM) expressed as signal/*μ*g of total protein (corrected for background signal) following exposure to either normoxia (control; FiO_2_ = 0.21, *n* = 6), hypoxia (FiO_2_ = 0.10, *n* = 7), or hypoxia + NAC (200 mg/kg i.p. at FiO_2_ = 0.10); ^∗^*p* = 0.0332, ^∗∗^*p* = 0.0019, and ^∗∗∗∗^*p* < 0.0001; unpaired *t* tests.

There was no significant difference in HIF-1*α* mRNA expression between groups (data not shown).  Figure 4 shows HIF-1*α* protein content in diaphragm muscle from mice exposed to 8 hours of normoxia, hypoxia, or hypoxia + NAC. Hypoxia significantly increased HIF-1*α* protein content compared with the control group (*p* = 0.0019; unpaired *t* test). HIF-1*α* protein content was further increased in hypoxia + NAC compared with normoxia (*p* < 0.0001) and was also significantly higher in hypoxia + NAC compared with the hypoxia group (*p* = 0.0332).

### 3.4. Cell Signalling: Akt Pathway

**Figure 5:**
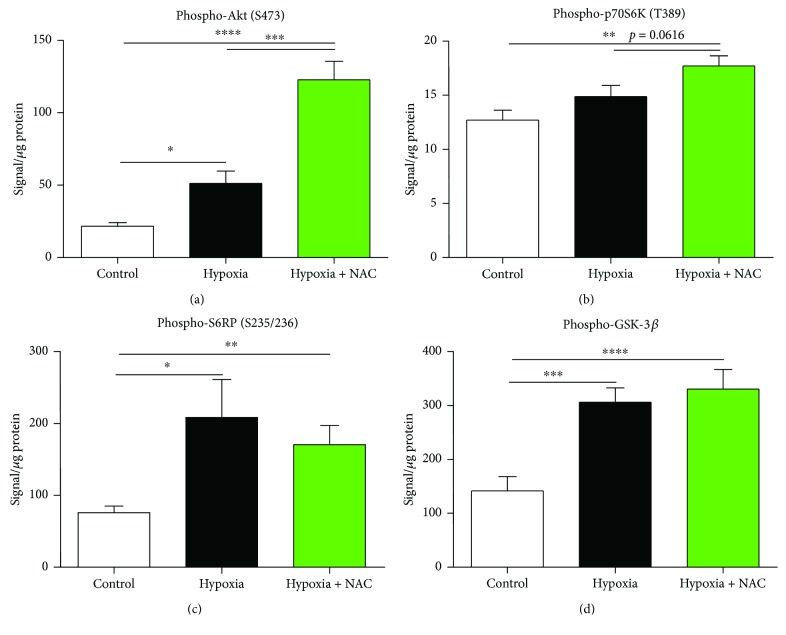
Mouse diaphragm phospho-Akt (S473), phospho-p70S6K (T389), phospho-S6RP (S235/236), and phospho-GSK-3*β* protein contents following 8 hours of exposure to normoxia (control), hypoxia, or hypoxia + NAC. (a) Diaphragm phospho-Akt (S473) protein content (mean ± SEM) expressed as signal/*μ*g of total protein (corrected for background signal); *n* = 8 per group. ^∗^*p* = 0.0109, ^∗∗∗∗^*p* = 0.0001, and ^∗∗∗^*p* = 0.0004; unpaired *t* tests. (b) Diaphragm phospho-p70S6K (T389) protein content (mean ± SEM) expressed as signal/*μ*g of total protein (corrected for background signal); *n* = 8 per group. ^∗∗^*p* = 0.0019; unpaired *t* test. (c) Diaphragm phospho-S6RP (S235/236) protein content (mean ± SEM) expressed as signal/*μ*g of total protein (corrected for background signal); *n* = 8 per group. ^∗^*p* = 0.0434 and ^∗∗^*p* = 0.0099; unpaired *t* tests. (d) Diaphragm phospho-GSK-3*β* protein content (mean ± SEM) expressed as signal/*μ*g of total protein (corrected for background signal); *n* = 8 per group. ^∗∗∗∗^*p* = 0.0006 and ^∗∗∗^*p* = 0.0009; unpaired *t* tests.

Phospho-Akt protein content in diaphragm muscle from mice exposed to 8 hours of normoxia, hypoxia, or hypoxia + NAC is shown in Figure 5(a). Exposure to hypoxia significantly increased phospho-Akt protein content compared with the control group (*p* = 0.0109; unpaired *t* test). Phospho-Akt protein content was further increased in hypoxia + NAC compared with normoxia (*p* = 0.0001) and was also significantly higher in hypoxia + NAC compared with the hypoxia group (*p* = 0.0004).

Figure 5(b) shows phospho-p70S6K protein content in diaphragm muscle from mice exposed to 8 hours of normoxia, hypoxia, or hypoxia + NAC. Exposure to hypoxia had no significant effect on phospho-p70S6K protein content compared with normoxia. However, hypoxia + NAC increased phospho-p70S6K protein content compared with the control group (*p* = 0.0019) and also increased phospho-p70S6K protein content above the level of the hypoxia group (*p* = 0.0616).

Figure 5(c) shows phospho-S6RP protein content in diaphragm muscle from mice exposed to 8 hours of normoxia, hypoxia, or hypoxia + NAC. Exposure to hypoxia significantly increased phospho-S6RP protein content compared with the control group (*p* = 0.0434). Hypoxia + NAC also increased phospho-S6RP protein content compared with the control group (*p* = 0.0099), but there was no difference between the hypoxia and hypoxia + NAC groups.

Figure 5(d) shows phospho-GSK-3*β* protein content in diaphragm muscle from mice exposed to 8 hours of normoxia, hypoxia, or hypoxia + NAC. Exposure to hypoxia significantly increased phospho-GSK-3*β* protein content compared with the control group (*p* = 0.0009). Hypoxia + NAC increased phospho-GSK-3*β* protein content further still compared with the control group (*p* = 0.0006), but there was no difference between the hypoxia and hypoxia + NAC groups.

### 3.5. Cell Signalling: mTOR Pathway

**Figure 6:**
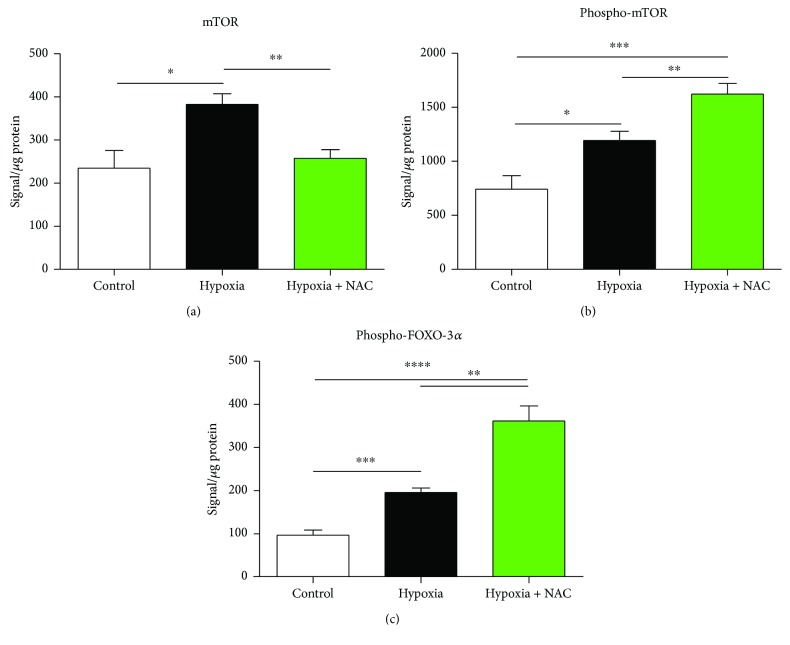
Mouse diaphragm mTOR, phospho-mTOR, phospho-ERK1/2, and phospho-FOXO3a protein contents following 8 hours of exposure to normoxia (control), hypoxia, or hypoxia + NAC. (a) Diaphragm mTOR protein content (mean ± SEM) expressed as signal/*μ*g of total protein (corrected for background signal); *n* = 7 − 8 per group. ^∗^*p* = 0.0107 and ^∗∗^*p* = 0.0020; unpaired *t* tests. (b) Diaphragm phospho-mTOR protein content (mean ± SEM) expressed as signal/*μ*g of total protein (corrected for background signal); *n* = 7 − 8 per group. ^∗^*p* = 0.0123, ^∗∗^*p* = 0.0067, and ^∗∗∗^*p* = 0.0001; unpaired *t* tests. (c) Diaphragm phospho-FOXO3a protein content (mean ± SEM) expressed as signal/*μ*g of total protein (corrected for background signal); *n* = 7 − 8 per group. ^∗∗^*p* = 0.0024, ^∗∗∗^*p* = 0.0002, and ^∗∗∗∗^*p* < 0.0001; unpaired *t* tests.

Figure 6(a) shows mTOR protein content in diaphragm muscle from mice exposed to 8 hours of normoxia, hypoxia, or hypoxia + NAC. Exposure to hypoxia significantly increased mTOR protein content compared with the control group (*p* = 0.0107). Hypoxia + NAC, however, did not increase mTOR protein content compared with the control group, and mTOR protein content was significantly lower in the hypoxia + NAC group compared with the hypoxia group (*p* = 0.0020).

Figure 6(b) shows phospho-mTOR protein content in diaphragm muscle from mice exposed to 8 hours of normoxia, hypoxia, or hypoxia + NAC. Exposure to hypoxia significantly increased phospho-mTOR protein content compared with the normoxia group (*p* = 0.0123). Phospho-mTOR protein content was further increased in the hypoxia + NAC group compared with the control group (*p* = 0.0001) and was also significantly higher than the hypoxia group (*p* = 0.0067).

### 3.6. Cell Signalling: FoxO3a Pathway

Figure 6(c) shows phospho-FoxO3a protein content in diaphragm muscle from mice exposed to 8 hours of normoxia, hypoxia, or hypoxia + NAC. Exposure to hypoxia significantly increased phospho-FoxO3a protein content compared with the normoxia group (*p* = 0.0002). Phospho-FoxO3a protein content was further increased in the hypoxia + NAC group compared with the control group (*p* < 0.0001) and was also significantly higher than the hypoxia group (*p* = 0.0024).

### 3.7. Cell Signalling: JNK, p38, and ERK1/2 Pathways

**Figure 7:**
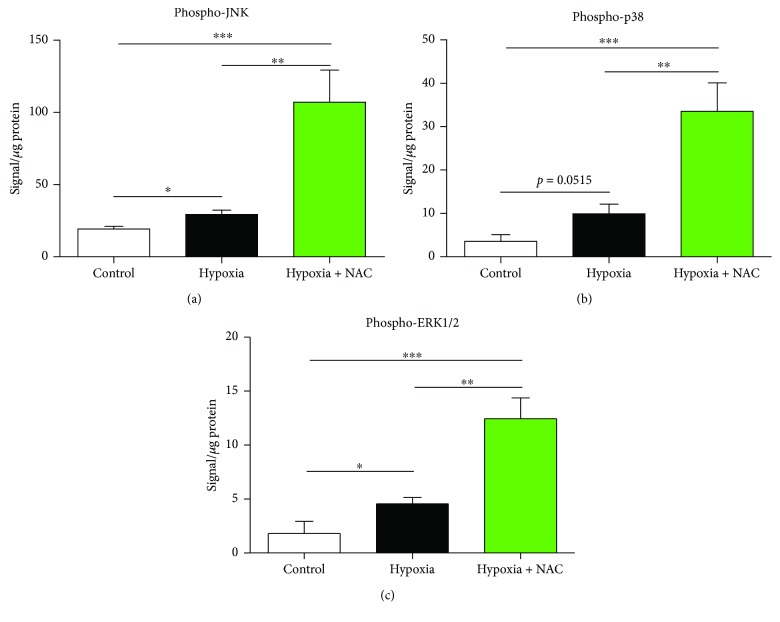
Mouse diaphragm phospho-JNK and phospho-p38 protein contents following 8 hours of exposure to normoxia (control), hypoxia, or hypoxia + NAC. (a) Diaphragm phospho-JNK protein content (mean ± SEM) expressed as signal/*μ*g of total protein (corrected for background signal); *n* = 8 per group. ^∗^*p* = 0.0120, ^∗∗^*p* = 0.0108, and ^∗∗∗^*p* = 0.0058; unpaired *t* tests. (b) Diaphragm phospho-p38 protein content (mean ± SEM) expressed as signal/*μ*g of total protein (corrected for background signal); *n* = 6–8 per group. ^∗∗^*p* = 0.0092 and ^∗∗∗^*p* = 0.0030; unpaired *t* tests. (c) Diaphragm phospho-ERK1/2 protein content (mean ± SEM) expressed as signal/*μ*g of total protein (corrected for background signal); *n* = 5–8 per group. ^∗^*p* = 0.0406, ^∗∗∗^*p* = 0.0020, and ^∗∗^*p* = 0.0046; unpaired *t* tests.

Phospho-JNK protein content in diaphragm muscle from mice exposed to 8 hours of normoxia, hypoxia, or hypoxia + NAC is shown in Figure 7(a). Exposure to hypoxia significantly increased phospho-JNK protein content compared with the normoxia group (*p* = 0.0120). Phospho-JNK protein content was further increased in the hypoxia + NAC group compared with the control group (*p* = 0.0058) and was also significantly higher than the hypoxia group (*p* = 0.0108).

Figure 7(b) shows phospho-p38 protein content in diaphragm muscle from mice exposed to 8 hours of normoxia, hypoxia, or hypoxia + NAC. Exposure to hypoxia increased phospho-p38 protein content compared with the normoxia group (*p* = 0.0515). Phospho-p38 protein content was further increased in the hypoxia + NAC group compared with the control group (*p* = 0.0030) and was also significantly higher than the hypoxia group (*p* = 0.0092).

Phospho-ERK1/2 protein content in diaphragm muscle from mice exposed to 8 hours of normoxia, hypoxia, or hypoxia + NAC is shown in Figure 7(c). Hypoxia significantly increased phospho-ERK1/2 protein content compared with the normoxia group (*p* = 0.0406). Phospho-ERK1/2 protein content was increased further in the hypoxia + NAC group compared with the control group (*p* = 0.0020) and was also significantly higher than the hypoxia group (*p* = 0.0046).

### 3.8. Atrophy and Autophagy Gene Expression

**Figure 8:**
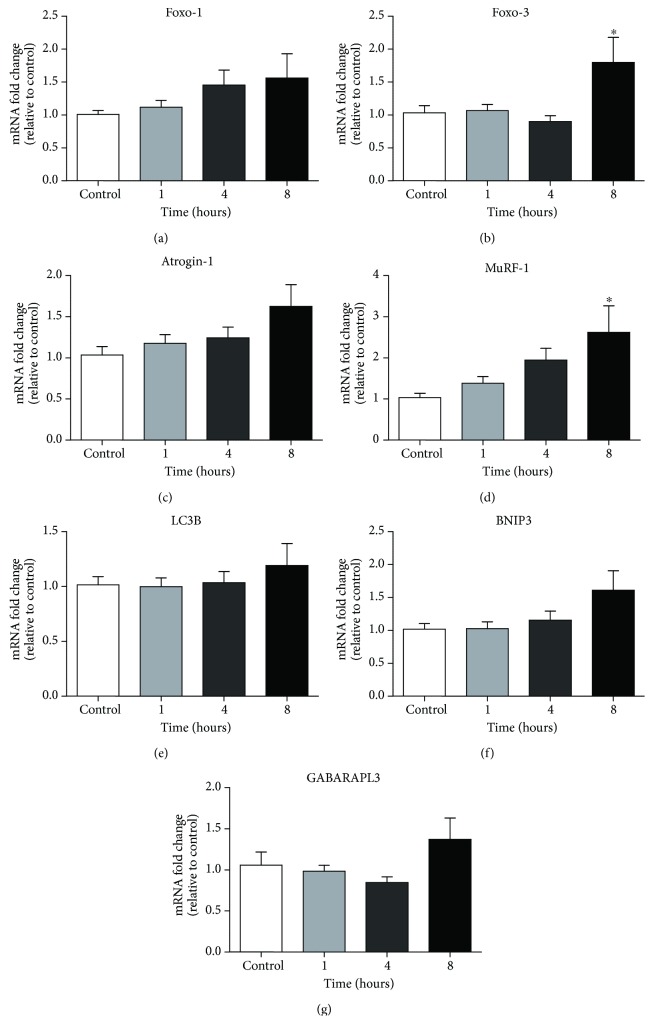
Mouse diaphragm muscle atrophy and autophagy gene expression following exposure to normoxia (control) or 1, 4, or 8 hours of hypoxia. Fold changes in mRNA expression (relative to the control (normoxia) group) for (a) FoxO-1; (b) FoxO-3; (c) Atrogin-1; (d) MuRF-1; (e) LC3B; (f) BNIP3; and (g) GABARAPL3; mean ± SEM, *n* = 7–8 per group. ^∗^*p* < 0.05 versus control, one-way ANOVA and Tukey's post hoc test following 8 hours of exposure to normoxia and 1, 4, and 8 hours of exposure to hypoxia.

Figure 8 shows expression data in diaphragm muscle for genes related to atrophy and autophagy. There were no significant changes in FoxO-1, Atrogin-1, LC3B, BNIP3, or GABARAPL3 mRNA expression between groups (one-way ANOVA and Tukey's post hoc test). FoxO-1, Atrogin-1, and BNIP3 displayed nonsignificant trends toward increased expression levels following increasing durations of hypoxic exposure (1, 4, and 8 hours). Both FoxO-3 and MuRF-1 mRNA expression was significantly increased following 8 hours of hypoxic exposure compared with control (*p* < 0.05, one-way ANOVA and Tukey's post hoc test) (Figures 8(b) and 8(d), resp.).

### 3.9. Oxidative Stress: TBARS Concentration

**Figure 9:**
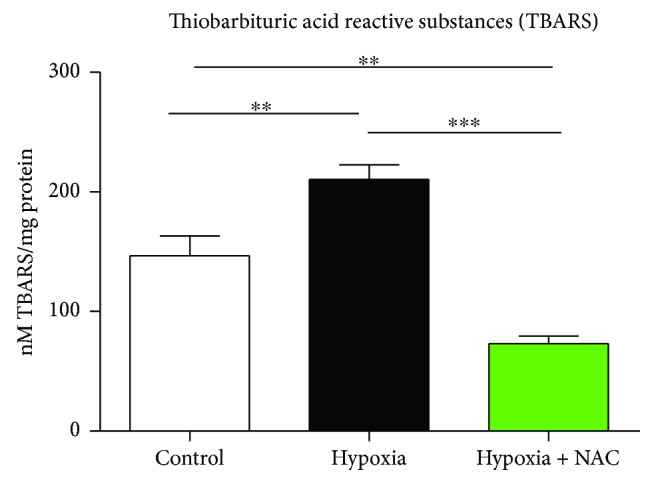
Mouse diaphragm muscle thiobarbituric acid reactive substances (TBARS) concentration following 8 hours of exposure to normoxia (control), hypoxia, or hypoxia + NAC. TBARS concentration in diaphragm muscle (mean ± SEM) expressed as nM TBARS per mg of protein following exposure to either normoxia (FiO_2_ = 0.21), hypoxia (FiO_2_ = 0.10), or hypoxia + NAC (200 mg/kg i.p. at FiO_2_ = 0.10); *n* = 8 per group. ^∗∗^*p* < 0.01 and ^∗∗∗^*p* < 0.0001; unpaired *t* test with Welch's correction.

Data for diaphragm TBARS concentration (a marker of lipid peroxidation) in normoxia, hypoxia, and hypoxia + NAC groups are shown in Figure 9. TBARS concentration was significantly increased following exposure to hypoxia compared with normoxia (*p* = 0.0076). TBARS concentration was significantly lower in NAC + hypoxia compared with hypoxia (*p* < 0.0001) and also significantly lower compared with the normoxia group (*p* = 0.0030).

### 3.10. Chymotrypsin-Like Proteasome Activity

**Figure 10:**
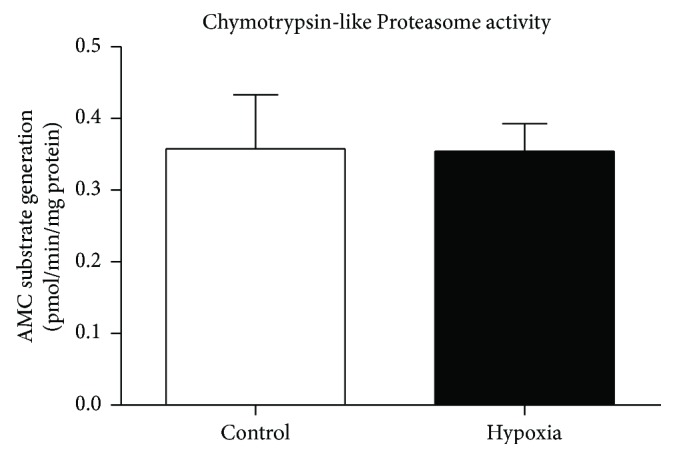
Mouse diaphragm muscle chymotrypsin-like proteasome activity following 8 hours of exposure to normoxia (control) or hypoxia. Chymotrypsin-like proteasome activity in diaphragm muscle (mean ± SEM) expressed as a rate of AMC tag release from peptide substrate (pmol/min/mg of protein) following exposure to either normoxia (control; FiO_2_ = 0.21) or hypoxia (FiO_2_ = 0.10); *n* = 8 per group. There was no significant difference between the two groups (unpaired *t* test).

Figure 10 shows diaphragm muscle chymotrypsin-like proteasome activity in normoxia and hypoxia groups. There was no significant difference in chymotrypsin-like proteasome activity between the two groups.

### 3.11. Diaphragm Muscle Fibre Size and Distribution

**Figure 11:**
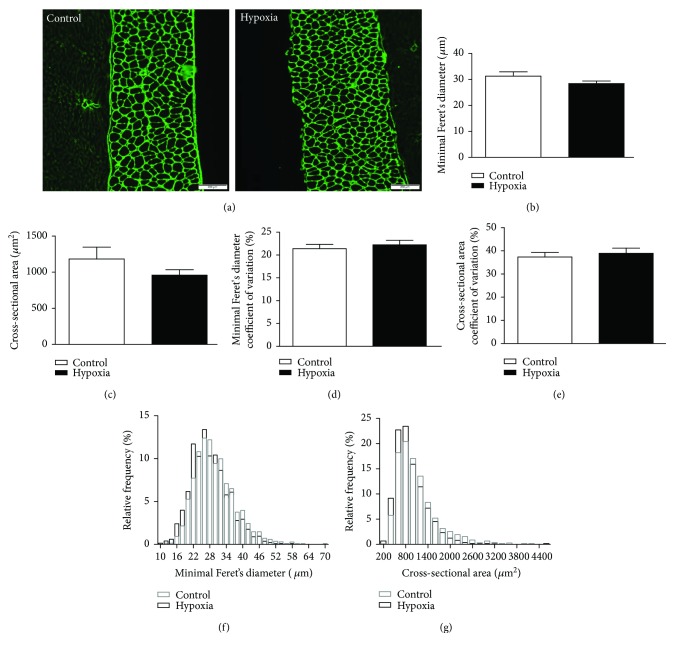
Mouse diaphragm muscle fibre size and distribution following exposure to 8 hours of normoxia (control) or hypoxia. (a) Representative images of mouse diaphragm muscle immunofluorescently labelled for laminin to highlight individual fibres from animals exposed to 8 hours of normoxia (control, FiO_2_ = 0.21) and hypoxia (FiO_2_ = 0.10). (b) Group data for mean minimal Feret's diameter. (c) Group data for mean muscle fibre cross-sectional area. (d) Group data for coefficient of variation of diaphragm muscle fibre size measured by minimal Feret's diameter. (e) Group data for coefficient of variation of diaphragm muscle fibre size measured by cross-sectional area. Data in (b–e) expressed as mean ± SEM; there were no significant differences between groups (*n* = 6 per group; unpaired Student's *t*-tests). (f) and (g) Relative frequency distribution of diaphragm muscle fibres measured by minimal Feret's diameter (f) and cross-sectional area (g).

Representative immunofluorescence images of diaphragm muscles from mice exposed to normoxia and hypoxia are shown in Figures 11(a) and 11(b). There was no significant difference in the mean minimal Feret's diameter (Figure 11(c)) or mean cross-sectional area of fibres (Figure 11(d)) between the two groups. The coefficient of variation of these indices was also equivalent in control and hypoxia muscles (Figures 11(e) and 11(f)). There was no shift in the relative frequency distribution of fibres in hypoxia compared with normoxia (Figures 11(f) and 11(g)).

## 4. Discussion

The main findings of this study are the following: (1) Acute hypoxic stress over several hours causes diaphragm muscle weakness; (2) Diaphragm dysfunction is unrelated to enhanced respiratory muscle work during hypoxic exposure, since ventilation is unchanged in mice exposed to sustained hypoxia; (3) Acute hypoxic stress stabilized diaphragm HIF-1*α* protein and activated MAPK, mTOR, Akt, and FoxO3a signalling pathways, favouring protein synthesis; (4) Autophagy gene expression was unaffected by acute hypoxia, but FoxO3 and MuRF-1 atrogene expression was progressively increased during hypoxic exposure without effect on diaphragm 20S proteasome activity; (5) Acute hypoxia increased diaphragm TBARS concentration indicative of oxidative stress; (6) Acute hypoxia did not affect diaphragm myofibre size or distribution; (7) NAC pretreatment strongly potentiated many of the endogenous cell survival signalling responses to acute hypoxic stress and reversed diaphragm oxidative stress; (8) Antioxidant pretreatment with NAC prevented acute hypoxia-induced diaphragm dysfunction.

### 4.1. Muscle Function

This study confirms our previous report [17], revealing that acute sustained hypoxia causes pronounced diaphragm muscle weakness. Our experimental approach was to study diaphragm performance ex vivo under standardized conditions. In this way, we determined that weakness is intrinsic to diaphragm muscle fibres, as we controlled the stimulation frequency. The approach allowed us to reestablish optimal oxygen and acid-base conditions for the study of muscle performance. Therefore, diaphragm function was assessed at normal pH, whereas the persistent hyperventilation associated with hypoxia exposure results in a respiratory alkalosis and elevated blood pH, which are likely to cause further perturbation to respiratory muscle function *in vivo*. Our data reveal that the force-generating capacity of diaphragm from hypoxic animals is intrinsically weak, especially at high stimulation frequencies, which correspond to activation of the muscle during airway protective behaviours [36]. Decreased peak force-generating capacity is linked to poor prognosis in respiratory patients [1]. Oxygen was provided abundantly in the ex vivo preparation allowing comparison of normoxia and hypoxia groups under similar conditions. We concede that whereas the hypoxia group was revealed as having decreased force-generating capacity compared with the normoxia group, we did not extend the study to determine how the hypoxia group would have compared with the normoxia group under conditions of bath hypoxia. For example, exposure to chronic sustained hypoxia has been shown to improve tolerance to a subsequent severe hypoxic stimulus [37] revealing that hypoxic adaptation in respiratory muscle can result in some protective outcomes relevant to the prevailing stimulus of oxygen deficit. Our data highlight the potential for acute hypoxic stress to adversely affect diaphragm performance, which has relevance to respiratory patients, particularly in the critical care setting [1], and may have implications for individuals at high altitude [19, 20]. We acknowledge that further assessments of diaphragm function *in vivo* are required, where respiratory muscle performance can be considered in the context of an integrative multisystem response to hypoxia (e.g., blood flow and neural drive), to lend further credence to our hypothesis that hypoxia is a driver of respiratory muscle dysfunction.

Antioxidant pretreatment with NAC prior to hypoxic exposure completely rescued the diaphragm from acute hypoxia-induced diaphragm weakness. Indeed, notwithstanding hypoxic exposure, NAC supplementation enhanced muscle force-generating capacity above control levels. The finding is reminiscent of a study showing that NAC treatment immediately after the initiation of 6 hours of mechanical ventilation completely prevented the development of mechanical ventilation-induced diaphragmatic weakness [8]. Of interest, in the latter study, NAC did not suppress autophagy, rather it augmented autophagosome formation suggesting that NAC exerts its beneficial effects on ventilator-induced diaphragm dysfunction via stimulation of autophagy (excess ROS can inhibit autophagy), which appears to be a beneficial adaptive response in the diaphragm to the physiological stress of mechanical ventilation, rather than representing a contributing factor to the development of diaphragm dysfunction [8]. NAC has also been shown to protect the diaphragm from the damaging effects of controlled mechanical ventilation including diaphragmatic oxidative stress and proteolysis, as well as controlled mechanical ventilation-induced contractile dysfunction [38]. We have previously reported beneficial effects of NAC in ameliorating diaphragm protein carbonylation, which presents progressively in response to exposure to chronic sustained hypoxia [16]. Indeed, protein carbonylation underpins the loss of skeletal muscle mass in a number of chronic conditions [39] suggesting that NAC and other antioxidants could have widespread utility as adjunctive therapies in skeletal muscle disease. Of interest, NAC also proved efficacious in preventing diaphragm muscle weakness and fatigue in a rat model of chronic intermittent hypoxia modelling sleep apnoea [32].

### 4.2. Metabolism

Acute hypoxic stress decreased metabolism in mice, as evident from the rapid and sustained reduction in CO_2_ production during hypoxia, as well as the reduction in postmortem body temperature following hypoxia. Interestingly, body temperature was reduced to an equivalent level in the hypoxia + NAC group revealing that the beneficial effect of NAC on hypoxic diaphragm performance is unlikely related to an influence on the hypoxic hypometabolic/hypothermic state and associated alkalosis caused by the reduction in CO_2_ production concomitant with maintained ventilation, that is, hypoxic hyperventilation. It is therefore conceivable to consider that the beneficial effects of NAC relate instead to direct antioxidant and/or signalling effects in muscle.

It is also noteworthy that minute ventilation was relatively unchanged over the 8 hours of hypoxic exposure in comparison with control animals. This reveals that diaphragm activity is not increased due to a hypoxia-induced increase in ventilatory drive (common to other mammals), and so increased muscle activity during hypoxic stress is not a contributory factor to the development of hypoxia-induced diaphragm weakness, although it may be a further aggravating factor during acute hypoxic stress in humans. Our study in mice reveals the potential for diaphragm muscle weakness related to direct hypoxic (redox) stress of muscle fibres [16]. The hypometabolic response causes a relative hyperventilation evident from the hypoxia-induced increase in the ventilatory equivalent for carbon dioxide. The ensuing alkalosis (which is achieved in humans during exposure to hypoxia by way of enhanced ventilation with no change in metabolism) may be a factor of relevance for diaphragm performance *in vivo*. Of interest, the respiratory exchange ratio was unchanged during exposure to hypoxia suggesting that there was not a hypoxia-induced switch in metabolic substrate preference away from lipids.

### 4.3. Hypoxia Signalling

HIF-1*α* protein content was increased in the diaphragm following 8 hours of hypoxia. This is not altogether surprising given that HIF-1*α* protein accumulation is indirectly proportional to cytosolic oxygenation, although muscle-specific and temporal responses have been reported in respiratory muscles in response to chronic sustained hypoxia [15, 16]. Reactive oxygen species can induce HIF-1*α* expression, but this effect can be dependent on exposure time, and reactive oxygen species can exert the opposite effect over prolonged periods, thus creating some uncertainty regarding how swings in cellular redox balance affect HIF-1*α* expression [40–43]. One might have expected that NAC, as an antioxidant, would influence hypoxia-induced increase in HIF-1*α* protein content. However, HIF-1*α* is responsive to redox balance within the intracellular environment and reducing conditions can stabilize HIF-1*α*. Moreover, NAC-induced increases in cellular glutathione can elevate HIF-1*α* expression [44–47]. In our study, pretreatment with NAC prior to hypoxic exposure enhanced HIF-1*α* protein content in diaphragm muscle compared with hypoxia alone. In view of the protective effect of NAC on diaphragm force-generating capacity, this suggests that HIF-1*α* stabilization was a component of the adaptive response of muscle to hypoxic challenge. Indeed, it has been reported that HIF-1*α* contributes to the neuroprotective effect of NAC in an ischaemic stroke model of transient cerebral ischaemia in rats [47].

### 4.4. Hypertrophy/Atrophy Signalling

**Figure 12:**
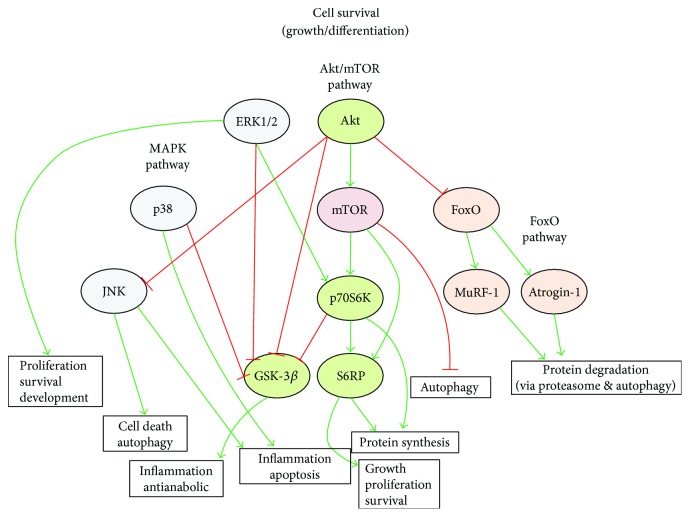
Schematic depicting the various cell survival signalling pathways explored in this study. Green arrows indicate activation, and red lines indicate inhibition in diaphragm muscle following exposure to acute hypoxic stress.

We examined the protein and/or phosphoprotein content of various proteins associated with signalling pathways involved in the balance between cell survival and death and protein balance (hypertrophy/atrophy) in diaphragm muscle (summarized in Figure 12).

### 4.5. Prohypertrophy/Cell Survival Signalling

#### 4.5.1. Akt Pathway

Akt, particularly the Akt/mTOR pathway, is central to the control of the hypertrophic/atrophic balance in muscle, acting in a phosphorylation-dependent manner as a key regulator of downstream effectors involved in modulating cell growth and survival. Activated (phosphorylated) Akt is predominantly prohypertrophic, via inhibition of atrophic signals and promotion of hypertrophic signals. In our study, phosphorylated (active) Akt (phospho-Akt) protein content was increased following acute hypoxia exposure and enhanced further still by NAC supplementation. It has been previously demonstrated that under certain conditions hypoxia can activate Akt and it is suggested that hypoxia-induced Akt activation may play a role in protection against apoptosis [48–51]. Similarly, in our model, it would appear that acute hypoxia-induced Akt activation is a beneficial compensatory mechanism supporting the functional integrity of the diaphragm muscle. NAC pretreatment enhanced the endogenous induction of Akt signalling, potentially bolstering the cellular defences against acute hypoxic stress. This may be (part of) the mechanism by which NAC rescues diaphragm force in acute hypoxia. It is also worth noting that Akt/mTOR signalling can regulate HIF-1*α* and may play a role in hypoxia-induced HIF-1*α* expression [52–54]. Akt also exerts prohypertrophic effects via inhibition of atrophic signalling (Figure 12).

Phospho-p70S6K, which is downstream of Akt, was slightly elevated by acute hypoxia in the diaphragm. Its protein content was, however, significantly elevated in the hypoxic diaphragm by NAC supplementation. p70S6k activation by Akt is mediated via the indirect activation of mTOR by Akt. p70S6K, when activated, promotes translation/protein synthesis and cell survival in muscle [55–57]. The fact that the induction of elevated phospho-p70S6K is greater in hypoxia + NAC compared with hypoxia alone is likely due to the greater elevation of phospho-Akt, phospho-mTOR, and phospho-ERK1/2 in hypoxia + NAC, which all activate phospho-p70S6K [58]. Again, this is indicative of prohypertrophy signalling induced by NAC supplementation, a mechanism which appears beneficial in the maintenance of diaphragm muscle performance. Indeed, decreased phospho-p70S6K expression occurs in the diaphragm of mechanically ventilated rats prior to decreased protein synthesis [59].

Phospho-S6RP content, which is a downstream of both Akt/mTOR and p70S6K, was significantly increased in the diaphragm both by hypoxia and hypoxia + NAC. The phosphorylation of S6RP by phospho-p70S6K, driven by Akt/mTOR, drives translation/protein synthesis and growth (Figure 12).

Hypoxia can activate Akt leading to GSK-3 phosphorylation [49]. Phosphorylation negatively regulates GSK-3*β*, inhibiting it, resulting in the activation of protein synthesis and a cessation of antianabolic signalling from GSK-3*β*. In our study, phospho-GSK-3*β* protein content was increased significantly by hypoxia and increased further still by hypoxia + NAC. Again, these data suggest a drive toward a prohypertrophic/proprotein synthesis state in acute hypoxia that is further augmented by NAC pretreatment.

#### 4.5.2. mTOR Pathway

mTOR is a protein kinase functioning in the regulation of growth, survival, and protein synthesis in skeletal muscle. Both mTOR and phospho-mTOR were measured in our study. mTOR mediates its hypertrophic/cell survival signalling effects via its phosphorylated/activated state. Acute hypoxia significantly increased mTOR protein content. Whereas hypoxia and hypoxia + NAC both increased phospho-mTOR protein content, expression was greatest in the hypoxia + NAC group. It appears that hypoxia alone, in the absence of NAC, induces an increase in mTOR protein expression, or indeed a reduction in mTOR degradation, while phosphorylation of mTOR is induced by hypoxia, and to a greater extent by hypoxia concomitant with NAC supplementation. Phospho-p70S6K and phospho-s6RP, discussed above, are also both downstream of, and activated by, mTOR, and so the increase in phospho-mTOR observed in our study likely contributes to the increases in phospho-p70S6K and phospho-s6RP reported above.

#### 4.5.3. MAPK Pathway

ERK1/2 is a member of the MAPK family which positively regulates p70S6K, negatively regulates GSK-3*β*, and itself positively regulates cellular proliferation, survival, and development. Through these mechanisms, it is procell survival/hypertrophy. Here, acute hypoxia increased diaphragm phospho-ERK1/2 protein content, and NAC pretreatment further augmented phospho-ERK1/2 protein content. This, combined with changes in Akt signalling described above, is further suggestive that NAC pretreatment bolsters the endogenous cellular signalling mechanisms acting to promote protein synthesis and cell survival in the acutely hypoxic diaphragm.

#### 4.5.4. FoxO3a Pathway

The FoxO pathway is generally associated with atrophy and protein degradation in its dephosphorylated state. However, when phosphorylated, FoxO3a is inhibited from translocation into the nucleus and exerting its atrophic effects. One mechanism by which Akt exerts a hypertrophic effect is by the phosphorylation and thus inhibition of FoxO3a, preventing the atrophic action of the atrogenes MuRF-1 and Atrogin-1, which would otherwise be transcriptionally upregulated by FoxO3a. Our study revealed that acute hypoxia increases phospho-FoxO3a protein content in the diaphragm and NAC pretreatment further augmented phosphoprotein content, thus enhancing the prohypertrophic/antiatrophic signalling cascades described above, via the downregulation of FoxO3a-induced proteasomal and autophagy-mediated protein degradation. The acute hypoxia-induced increase in phosphoprotein content is likely mediated via the increase in phospho-Akt.

### 4.6. Proatrophy/Cell Death Signalling

#### 4.6.1. MAPK Pathway

While there appears to be a predominant drive toward protein synthesis in the acutely hypoxic diaphragm, augmented further by NAC pretreatment, there is also an increase in atrophic signalling via the MAPK pathway. JNK promotes autophagy, apoptosis, inflammation, and cell death and is activated by phosphorylation [60]. We observed that acute hypoxia elevated diaphragm levels of phospho-JNK protein, an effect which is further augmented by NAC supplementation. This is somewhat surprising given that Akt, which is also activated, negatively regulates the JNK pathway via a cross-talk mechanism [61]. JNK phosphorylation in our model is therefore likely induced by some other mechanism/stress affecting the MAPK pathway. p38 can promote inflammation and apoptosis, while also negatively regulating GSK-3*β* to promote survival [62]. In our study, acute hypoxia increased diaphragm phospho-p38 expression to near significant levels in the diaphragm, while NAC concomitant with acute hypoxia significantly augmented this response. This suggests a proatrophic effect of hypoxia + NAC in the diaphragm. We acknowledge that our assessment of apoptotic pathways is incomplete.

We observed no change in autophagy gene expression. We do not have information on autophagy signalling at the protein level, but this is an area worthy of further pursuit. We observed no change in the expression of several atrogenes, but there was an increase in the mRNA expression of FoxO3 and MuRF-1 in the diaphragm after 8 hours of hypoxia exposure. Despite this apparent increase in atrophy signalling, there was no downstream change in diaphragm proteasome enzymatic activity, suggesting that protein catabolism did not occur, at least not within 8 hours of hypoxic stress, which suggested that diaphragm muscle weakness in hypoxia is not likely related to muscle fibre atrophy, a phenomenon that has been observed in respiratory muscles following chronic sustained hypoxic exposure [13, 14]. Immunohistochemical analysis of diaphragm muscle fibre size and distribution revealed that acute sustained hypoxia did not cause myofibre atrophy or a shift in the size distribution of fibres. Therefore, we conclude that myofibre contractile dysfunction underpins diaphragm weakness following acute hypoxia, a phenomenon that has been reported, in addition to fibre atrophy, in diaphragm in response to chronic sustained hypoxia [63].

We employed a TBARS assay as a measure of muscle lipid peroxidation. We acknowledge the limitation of this measurement as an index of oxidative stress. Malondialdehyde is generated only by certain lipid peroxidation products, and it is not generated exclusively by lipid peroxidation. Exposure to acute hypoxia resulted in a modest but significant increase in diaphragm malondialdehyde, which we interpret as indicative of oxidative stress, but this needs to be confirmed in future studies. Although the source of reactive oxygen species was not determined in our study, the inhibitory effects of reactive oxygen species on striated muscle force-generating capacity are well described [64–66]. Pretreatment with NAC completely reversed the hypoxia-induced increase in diaphragm lipid peroxidation, decreasing TBARS levels to below control values. This reveals the antioxidant capacity of NAC and suggests that some of its inotropic action in diaphragm muscle exposed to hypoxia most likely related to suppression of hypoxia-induced oxidative stress. We acknowledge that the effects of NAC pretreatment were not determined in all experiments.

## 5. Conclusions

Figure 12 provides an overview of the effects of acute hypoxia on diaphragm protein signaling pathways pivotal to protein turnover and cell survival. Our study determined that acute hypoxic stress of just several hours is sufficient to cause diaphragm weakness, which may have clinical relevance. Hypoxia-induced diaphragm dysfunction is prevented by NAC pretreatment, via a mechanism independent of hypoxic hypometabolism in mice. We previously demonstrated that exposure to acute hypoxia increases UCP-3 mRNA expression in the mouse diaphragm suggesting an increased reliance on fatty acid metabolism [17, 18]. However, in the present study, we determined that the respiratory exchange ratio is unaffected by acute hypoxia, suggesting that there is no switch in metabolic energy source; the ratio of 0.7 reveals that fatty acids are the primary substrate for oxidative metabolism and energy production in normoxic mice, and this is unaltered by hypoxic exposure. This differs from the pronounced metabolic remodelling that occurs in respiratory muscles exposed to chronic sustained hypoxia [15, 16]. The hypoxia-induced increase in UCP-3 [17] may therefore be a compensatory mechanism to reduce ROS production and oxidative stress. Nevertheless, acute hypoxia results in diaphragm muscle oxidative stress, which is likely implicated in the elaboration of diaphragm weakness. Hypoxia did not cause any persistent change in ventilation (diaphragm muscle activity), and therefore muscle weakness and molecular changes are likely directly related to hypoxic stress per se and unrelated to altered mechanical work in hypoxia, though this may be a factor in other mammals such as humans. NAC pretreatment potentiates endogenous cell survival signalling and prevents hypoxia-induced diaphragm oxidative stress. The drive to increase both hypertrophy and atrophy signalling may be required to increase protein turnover and amino acid-dependent ATP generation, but notably diaphragm myofibre size and distribution are unaffected. NAC appears to boost endogenous cell survival signalling cascades potentiating defence responses, perhaps in this way preserving diaphragm function in the face of redox stress, in addition to established antioxidant actions. Our findings highlight the potentially critical role of hypoxic stress as a contributor to diaphragm myofibre dysfunction in respiratory patients. Our results may also have relevance to respiratory muscle performance at high altitude. On the basis of our findings in an animal model, we conclude that NAC may be a beneficial adjunctive therapy in patients, alleviating hypoxia-induced diaphragm dysfunction, potentially improving patient outcome.
